# Supplementary material for: Cardiac Toxicity Associated with Immune Checkpoint Inhibitors: A Systematic Review
Source: Int J Mol Sci. 2022 Sep 19;23(18):10948. doi: 10.3390/ijms231810948 (PMC9502843; doi:10.3390/ijms231810948)
Supplement: Supplementary file 1 [file ijms-23-10948-s001.zip › ijms-1892675-supplementary.pdf]

Table S1. ICI associated myocarditis.

Study	Patient characteristics	Medical history	Symptoms	Diagnosis	CV side effect	ICI	Type of cancer	Myocarditis onset	Myocarditis treatment	Evolution
Ida, 2022	♀, 81	HBP dyslipidaemia	high-grade fever<br>whole-body rash<br>altered consciousness	↑CK; ↑CKMB , troponin I,C-reactive protein, ECG, TTE<br>CMR<br>endomyocardial biopsy	Myocarditis	PD-L1i + PD-1i	advanced melanoma,	One wwk	methylprednisolone followed by prednisolone<br>IGIV	favorable
Nguyen, 2022	♂, 25	N/A	chest pain, subtle myalgia	Coronary angiography<br>cardiac MRI<br>endomyocardial biopsy<br>Troponin-T<br>creatine-kinase	Myocarditis<br>myositis	PD-1i	thymoma	Two week	Methylprednisolone<br>mycophenolate-mofetil<br>loading dose of intravenous abatacept<br>oral ruxolitinib	cardiogenic shock<br>VT<br>extracorporeal life support<br>On day 40, the patient fully recovered clinically
Okauchi, 2022	♂, 60	Smoking history	Cough, dyspnea	Chest RX<br>BNP<br>TTE	Myocarditis	PD-1i	Squamous cell carcinoma	130 weeks from initiation	Diuretics, beta-blocker	Favorable
Zhou, 2022	♂, 67	N/A	fever, chest pain and dyspnea	Chest computed tomography, TTE , ECG, BNP, troponin T , CK	myocarditis	PD-L1i	lung squamous cell carcinoma stage IV	days after last cycle	methylprednisolone	favorable
Zhao, 2022	♂, 60	N/A	fever, tachycardia, hypotension, fatigue, dyspnea	ECG,<br>TTE	Myocarditis + hypothyroidism	PD-1i	soft tissue sarcoma	8 weeks	IV mPSL	favorable

Lorente-Ros, 2022	♂, 70	nephrectomy	2 episodes in the previous 12 hours of severe dizziness, dyspnea, and profuse sweating.	ECG troponin I,C-reactive protein, TTE ECG- 3rd AVB, coronary angiography, brain CT autoimmunity lab tests, brain, MRI , lumbar puncture , EEG	Myocarditis + Encephalitis	PD-1i + CTLA-4i	renal cell carcinoma	19 days	Temporary pacemaker high-dose iv corticosteroids intravenous immunoglobulins	delirium deterioration in his level of consciousness, intubation. Extubate -> reintubation on discharged
Saishu 2022	♀, 55	N/A	quadrantanopia, ocular motility disorder, diplopia, dysphagia, ocular motility disorder, muscle weakness of the extremities, bilateral ptosis muscle weakness.	↑CK; ECG, TTE; anti-AchR ab.	Myocarditis, myositis/MG	PD-1i	mela-noma	2 weeks	IGIV, prednisolone Intubation for MV, mPSL plasma exchange Tracheostomy	favorable
Yang, 2022	♀, 51	contrast agent allergy	high fever, mild dyspnea, and systemic rash.	Liver function indexes cardiac markers CT examination	myocarditis hepatitis	PD-1i	breast cancer (TNBC)	Three days	Iv methylprednisolone **Antibiotics hepatic protectors**	favorable
Ederhy, 2021	♀, 60-70 approx.	N/A	diplopia	↑TnI; ECG; CMR, coronary angiography, EMB	Myocarditis (subclinical)	PD-1i	Lung cancer (unmentioned type, metastatic)	3 infusions + 10 days	steroids, plasmapheresis	favorable
Tsuruda, 2021	♂, 75	N/A	asymptomatic	↑cTnT, CK, CK-MB; ECG, Echocardiogram, CMR, EMB	Myocarditis (subclinical), TTS	PD-1i	NSCLS (squamous, recurrent)	3 weeks	mPSL	fatal
	♂, 47	N/A	asymptomatic	↑cTnT, CK, CK-MB; CMR	Myocarditis (subclinical)	PD-1i	Ethmoid sinus cancer	3 infusions + 16 days	mPSL; IVIG (progressive thrombocytopenia); cyclosporine (hemophagocytic syndrome)	favorable
	♂, 63	N/A	hypotension (84/42 mmHg), tachycardia (132 bpm), tachypnea	↑cTnT, CK, AST, ALT, CRP, Cr;	cardiac complication of	PD-1i	Hypopharyngeal cancer	5 infusions + 32 days	cardioversion, extracorporeal	favorable

			(22 rpm); high fever, decreased appetite,	↓ WBC, Hb, PLT; ECG, Echocardiogram	cytokine-releasing syndrome				hemoperfusion with polymyxin B + continuous hemodiafiltration, catechocardiography lamines, broad-spectrum antibiotics, recombinant thrombomodulin, IVIG, high-dose corticosteroids	
Tanabe, 2021	♂, 75	N/A	posterior neck pain, neck drop	↑TnI, CK, CK-MB; ↑Eo (834/μL), ↓eRFG; DLST (+); Echocardiogram, coronary angiography, CMR,	Myocarditis (subclinical)	PD-1i + CTLA-4i	RCC (clear cell, metastatic)	53 days	prednisolone	favorable
Barham, 2021	♀, 79	N/A	dizziness, abdominal bloating, hypoxic	↑LDH; ECG; EMB	Myocarditis (grade 4); hyperprogression	PD-1i + CTLA-4i	Melanoma (vaginal, metastatic)	23 days	steroids; carboplatin + paclitaxel (salvage therapy), atropine, pacemaker (for AVB III)	fatal
Xie, 2021	♂, 67	N/A	exertional dyspnea, ptosis, blurred vision, quadriparesis	↑TnI, CK, CK-MB, AST, ALT, BNP, Mb; ECG; Echocardiogram; coronary angiography	Myocarditis (fulminant), MG crisis, hepatic dysfunction; delayed ir pneumonitis	PD-1i + pemetrexed + carboplatin	LCNEC (metastatic)	2 weeks	mPSL; pacemaker (temporary → permanent); ganciclovir/cefmetazole	favorable
Hu, 2021	♂, 63	N/A	chest tightness, limb weakness, dorsal myasthenia, diplopia, dysphagia	↑Hs-TnI, CK-MB, NT-proBNP, CK; Echocardiogram, CMR; anti-β1AR ab, CC ab, anti-myosin heavy chain ab,	Myocarditis + MG	PD-1i	ureteral urothelial cancer IV	3 weeks	mPSL IVIG	favorable

				ribonucleoprotein ab						
Winter sperge r, 2021	♂, 52	N/A	fatigue dyspnea	↑hsTnI, CK, BNP; ECG, Chest CT, Echocardiogram, CMR, EMB	Myocarditis	PD-L1i + investigatio nal ICI	melanoma	3 weeks	mPSL prednisone infliximab IV MMF	favorable
	♀, 60	N/A	general- ized weakness muscle pain fatigue fever	↑CK, hsTnI; ECG, Coronary angiography, CMR, EMB	Myocarditis	PD-L1i	gynecological cancer	2 weeks	mPSL prednisone	favorable
	♀, 49	N/A	fever cough	↑hsTnI, BNP; chest CT, ECG, CMR	Myocarditis	PD-L1i	triple- negative breast cance	2 weeks	MMF prednisone	favorable
	♀, 74	N/A	general pain, progressive muscle weakness diplopia	↑hsTnI, BNP, ECG, Coronary angiography, CMR	Myocarditis	PD-L1i	gynecological cancer	2 weeks	mPSL prednisone MMF	favorable
Stein- Merlo b 2021	♀, 60	N/A	palpitations reduced exercise tolerance, cool extremities altered mental status	↑Tn, BNP; ECG, Echocardiogram, Coronary angiography, CMR	Myocarditis Ocular myasthenia, colitis hepatitis	PD-1i	Colon cancer		Metoprolol succinate, lisinopril Continued immunosuppression spironolactone, Oral amiodarone, wearable defibrillator.dopami ne Nitroprusside milrinone, VA-ECMO	favorable
Shen 2021	♀, 53	N/A	cough chest congestion, muscle weakness fatigability drooping eyelids,	↑CK, CK-MB; ECG	Myocarditis, hepatitis, renal dysfunction, hypothyroidism	PD-1i + paclitaxel + platinum	type B3 thymoma	3 weeks	Magnesium isoglycyrrhizinate reduced glutathione injections, prednisone mPSL euthyrox pyridostigmine	favorable

Miyauchi 2021	♂,71	hypertension, DM2, hyperuricemia	Asymptomatic, chest tightness, shortness of breath, cardiogenic shock	↑CK, CM-MB, TnI, NT-proBNP; ECG, catheterization, EMB, CMR	Myocarditis	CTLA-4i + PD-1i	RCC	8 weeks	dopamine, dobutamine, noradrenaline intra-aortic balloon pump was inserted, adaptive servo ventilation mPSL prednisolone	favorable
Luo 2021	♀, 47	N/A	diplopia, myalgia, limb weakness, dysphagia, dyspnea	↑TnI, CK, ECG, EMG; RyR-ab, AChR-ab, anti-fibrillarin ab, anti-NOR-90 ab anti-Ro-52 ab	Myocarditis, myositis, MG	PD-1i	thymoma	3 weeks	neostigmine IVIG mPSL prednisolone pacemaker	favorable
Li 2021	♂, 62	hypertension, coronary heart disease	fever lethargy, cognitive dysfunction tachypnea hypoxia hypotension oliguria	↑Mb, Tn, CK-MB; ECG	cardiotoxicity kidney toxicity.	PD-1i	lung adenocarcinoma	48 weeks	mPSL continuous renal replacement therapy	favorable
Jespersen 2021	♂, 57	N/A	headache myalgia, palpitations binocular diplopia, ptosis, muscle weakness	↑TnI, CK-MB, Mb, CK; ECG, EMG, Echocardiogram, CMR, AChR-ab,	Myocarditis + myositis	CTLA-4i + PD-1i	RCC	2 weeks	temporary pacemaker. mPSL abatacept MMF implantable cardio-defibrilator	favorable
Iwasaki 2021	♀, 70	hypertension, aortic stenosis, chronic renal failure	shortness of breath fatigue	↑CK, CK-MB, TnT, NT-proBNP; ECG, Echocardiogram, CMR, Cardiac catheterization, EBM Coronary angiography	Myocarditis + myositis	PD-L1i	HCC	<1 week	cariperitide, furosemide, mPSL prednisolone	favorable
Hernández 2021	♀, 48	N/A	shortness of breath, dyspnea, bilateral ptosis blurred vision	↑Hs-TnI, NT-proBNP, CRP, CK; ECG,	Myocarditis + myositis (MG)	PD-1i	thymoma	<2 weeks	IV isoproterenol drip mPSL Infliximab temporary	fatal

				Echocardiogram, Coronary angiography, EMB; AChR-ab					pacemaker, dual-chamber pacemaker. intravenous amiodarone, noradrenaline dobutamine, intravenous anti-thymocyte globuline, pyridostigmine, ECMO	
Giblin 2021	♀, 47	N/A	dermatitisdiarrhea, palpitations	↑hS-TnI, BNP, Echocardiogram, CMR, Coronary angiography, EMB	Myocarditis (subclinical)	CTLA-4i + PD-1i	melanoma	1 week	mPSL prednisolon, IVIG	favorable
Cao 2021	♂, 69	N/A	ptosis, diplopia, shortness of breath,	↑CK, CK-MB, Mb, hs-TnT, NT-proBNP, LDH, ECG; Echocardiogram, EMG	Myositis, Myocarditis SJS/TEN	PD-1i	esophagogastric junction carcinoma	2 weeks	mPSL IVIG plasmapheresis	favorable
Ai, 2021	♂, 72	N/A	asymptomatic	ECG; CMR	Myocarditis (DRESS)	PD-1i	gastric adenocarcinoma	3 weeks	SCS (for DRESS)	
Liu and Jiang, 2020	♀, 68	N/A	dyspnea, fatigue	↑TnT, CK, CK-MB, NT-proBNP; ECG; CMR; ANA (1:1000)	Myocarditis (grade 3)	Sintilimab (PD-1i)	Stage IV Breast cancer (stage IV) + Hodgkin lymphoma	3 weeks	mPSL, PLEX, tofacitinib; diuretics, β-blocker, ACEi,	favorable
	♀, 46	N/A	dyspnea, palpitations	↑TnT, CK, CK-MB; CMR; ANA (1:1000)	Myocarditis	PD-1i	Supraclavicular lymph node metastasis with unknown primary site	30 weeks	mPSL, IVIG, tofacitinib	favorable

Jacob, 2020	♀, 66	N/A	Chest pain	↑TnT, TTE, GLS , CT chest angiogram Serial hs TnT Coronary angiography – no obstructive MRI, endomyocardial biopsy	Myocarditis	PD-L1i	Low grade follicular lymphoma	One month	PD-L1i Therapy stop Iv corticosteroid -> oral	favorable
Liu, 2020	♀, 78	Hypertension, DM2, hyperlipidemia	exertional dyspnea, irregular HR (105 - 140 bpm), HF signs; asymmetric ptosis, proximal muscle weakness, dysphagia	↑hs-TnT, CK, NT-proBNP; ECG; echocardiogram, coronary angiography, CMR (unsuggestive)	Myocarditis, neuromuscular complications	PD-1i	Melanoma (stage III)	5 weeks	mPSL, MMF, abatacept, plasmapheresis; amiodarone (monomorphic VT), shocks + temporary pacemaker (R-on-T VT, high-grade AVB), spironolactone + furosemide (HFpEF), bisoprolol (atrial tachycardia), oxygen therapy	fatal
Hardy, 2020	♂, 81	Typical lung carcinoid tumor (T1aN0), no autoimmune disease	fatigue, decreased appetite, weight loss	↑Tn, CK; ECG; cardiac catheterization; anti-striated muscle ab, anti-GM1 ab	Myocarditis	PD-1i + CTLA-4i	RCC (clear cell with sarcomatoid component, metastatic)	first infusion (< 21 days)	mPSL, plasmapheresis; aspirin, temporary pacing (AVB III)	fatal
Mirabel, 2020	♂, 57	N/A	myalgias, dyspnea	↑hs-TnI, BNP; ECG; cardiac catheterization, CMR, EMB	giant cell Myocarditis	PD-1i	RCC (metastatic)	78 weeks	mPSL; ACEi, BB	favorable
Ansari-Gilani, 2020	♂, 83	Hypertension, hypothyroidism	abdominal pain; unilateral facial drop, diffuse weakness	↑Tn; ECG; Echocardiogram, CMR, cardiac catheterization	Myocarditis; hepatitis, Bell's palsy, myositis, encephalitis	PD-1i	RCC	4 weeks	steroids	fatal

	♀, 78	Hyperlipidemia, depression/anxiety	malaise, blurry vision, dysphagia	↑Tn; ECG; CMR, cardiac catheterization	Myocarditis; MG, myositis, conjunctivitis/uveitis, hepatitis	PD-1i + CTLA-4i	Melanoma (metastatic)	18 days	steroids, PLEX	fatal
	♂, 81	Hypertension, COPD, AF	chest pain, watery diarrhea	↑Tn; ECG; Echocardiogram; CMR	Myocarditis; colitis	PD-1i → PD-1i + CTLA-4i	Melanoma (metastatic)	25 days	steroids	favorable
Loong Tan, 2020	♂, 62	Smoking, RBBB, LAFB	nausea, vomiting, chest pain	↑hsTnT, NT-proBNP; ECG; Echocardiogram, coronary angiography, CMR	myopericarditis, TTS, possible ICI-related pneumonitis	PD-1i	HCC (metastatic, HCV cirrhosis Child-Pugh B)	3 weeks	mPSL; broad spectrum antibiotics	favorable
Balanescu, 2020	♂, 75	N/A	cough, dyspnea; fever, rash	↑cTnI, BNP; ECG, echocardiogram; CMR, coronary angiography, EMB	myopericarditis	CTLA-4i + PD-1i	Myelodysplastic syndrome	33 days	IVIG, rosuvastatin	favorable
	♂, 78	N/A	fever, pneumonia	↑cTnI, BNP; ↓PLT; CMR, coronary angiography, EMB	myopericarditis	CTLA-4i	Myelodysplastic syndrome	6 days	IVIG, colchicine, atorvastatin	favorable
	♀, 74	N/A	dyspnea, hypotension; fever, rash	↑cTnI, BNP; ECG; CMR (unsuggestive), coronary angiography, EMB	Myocarditis	CTLA-4i	Melanoma (metastatic)	4 weeks	IVIG, colchicine, rosuvastatin, HCQ (for severe rash and arthralgias)	fatal
Yanase, 2020	♂, 59	N/A	bilateral ptosis, malaise, gaze palsy, impaired taste	↑TnI, CK, CK-MB; ECG; coronary angiography; anti-AchR ab	Myocarditis, MG	PD-1i + CTLA-4i	RCC (metastatic)	3 weeks	mPSL, PLEX, IVIG	favorable
Shalata, 2020	♀, 78	Hypertension, hypothyroidism	chest pain	↑TnI; Echocardiogram; CMR, cardiac catheterization, EMB	Myocarditis	PD-1i	Melanoma (metastatic)	40 days	prednisone	favorable

	♀, 55	Hypertension, DM2, post-thyroidectomy (papillary carcinoma)	chest pain, dyspnea; headache, fever, weakness; skin rash, pruritus	↑Tn, Echocardiogram; CMR (unsuggestive), coronary angiography	Myocarditis	PD-1i → PD-1i + CTLA-4i	Melanoma (metastatic)	13 weeks	prednisone	favorable
Leaver, 2020	♂, 55	Hypertension, smoking (previous), no family history of CAD	blurred vision, mild bilateral ptosis, fatigable left arm abduction	↑hs-TnI, CK; ECG; CMR, coronary angiography	Myocarditis (subclinical), MG, hepatitis, thyroiditis	PD-1i + CTLA-4i	Melanoma (metastatic)	4 weeks	prednisone, IVIG, MMF, aspirin (for CAD)	favorable
Diamantopoulosa, 2020	♀, 66	DM2, hypertension, hyperlipidemia, thyroidectomy, hypothyroidism (history of autoimmune disorder)	exertional dyspnea, orthopnea; diplopia, unilateral ptosis, gaze palsy, hypernasal speech, dysphagia for liquids	↑hs-cTnT, CK-MB; Echocardiogram; coronary angiography; anti-AchR ab	MG with cardiac involvement	CTLA-4i	Melanoma (metastatic)	15 days	pyridostigmine, prednisolone	favorable
	♂, 68	N/A	bilateral limb edema; face paresthesia, mild hypophonia, dysphagia, bilateral ptosis, diplopia	↑hs-cTnT, CK-MB; coronary angiography; anti-AchR ab	MG with cardiac involvement	PD-1i	Lentigo melanoma (metastatic)	3 weeks	pyridostigmine (discontinued due to bradycardia), prednisolone, IVIG	favorable
	♀, 80	DM2, hypertension	rapidly worsening fatigue, bilateral ptosis, gaze palsy, muscle weakness, distal loss of proprioception and vibration at lower limbs, dysphagia	↑hs-cTnT, CK-MB; Echocardiogram; anti-GM1 ab	Myocarditis, axonal polyradiculoneuropathy	PD-1i + CTLA-4i	Melanoma (stage IIIB)	3 weeks	mPSL, IVIG	favorable
Jeyakumar, 2020	♂, 86	CAD (4-vessel by-pass), SSS after pacemaker	decreased left vision, severe fatigue, lower back and bilateral hip pain, ptosis, difficulty arising from chair, muscle weakness, dysphonia,	↑hs-TnT, TnI, CK, CK-MB, ↑urinary Mb; ECG; EMB;	Myocarditis, myositis, MG	Cemiplimab (PD-1i)	SCC (recurrent, metastatic)	3 weeks	mPSL, PLEX, IVIG; intubation, renal replacement therapy	fatal

		placement, hypertension, hyperlipidemia, CKD, history of cutaneous carcinomas (basal cell, spindle cell, squamous cell)	pharyngeal secretions, dysphagia, dyspnea	anti-SM ab, anti-AchR ab, anti-Ro ab					
Doms, 2020	♂, 57	N/A	dyspnea, chest pain; weakness of the lower limbs, ptosis, diplopia	↑hs-TnI, TnT, CK, ECG; coronary angiography	Myocarditis	PD-1i + CTLA-4i	SCLC (neuroendocrine, metastatic)	2 infusions	mPSL, tocilizumab; pacemaker	favorable
Norwood 2020	♀, 57	No CV risk factors	Nausea	cTnI, CK ECG, TTE, cardiac MRI Endomyocardial biopsy PET CT , GLS	Thyroiditis Myocarditis	PD-1i + CTLA-4	Melanoma	2 weeks	Methylprednisolone Prednisone Single dose infliximab Tacrolimus Anti thymocyte globuline	After seven month – death – no myocarditis – post mortem examination Metastasis liver, spleen, bones
Catatonias, 2020	♀, 30	N/A	severe dyspnea, decreased exercise capacity, tachycardia, hypotension (BP = 90/70 mmHg), tachypnea (RR = 25 rpm), bibasilar pulmonary rales, lower limb edema	↑hs-TnT, NT-proBNP; ECG; Echocardiogram; CMR, coronary angiography, EMB	Myocarditis, HF	PD-1i	NSCLC (adenocarcinoma, metastatic)	6 infusions	mPSL, prednisolone, MMF; inotropes; bilateral thoracentesis, furosemide, spironolactone; amiodarone, lidocaine, magnesium, temporary TVP → MR-conditional ICD	favorable

									(symptomatic VT); ARB , BB, diuretics	
Iniesta, 2020	♂, 69	Urothelial bladder cancer (complete remission 2 years prior)	sudden episode of dyspnea, tachypnea (RR = 35 rpm), intercostal retraction, raised jugular venous pulse, bilateral pulmonary rales, tachycardia (110 bpm), hypertension (165/90 mmHg)	↑hs-cTnT, CK; ECG; echocardiogram; CMR, coronary angiography	Myocarditis, autoimmune hepatitis, myasthenia-like syndrome	PD-1i	NSCLC (type not mentioned, metastatic)	25 days	mPSL; O2 therapy, furosemide, sodium nitroprusside; enalapril, bisoprolol, spironolactone	favorable
Xing, 2020	♂, 66	Smoking (30 years), MG, radical resection of type AB thymoma, left lung adenocarcinoma	fatigue, myalgia and tender muscles in extremities, progressive muscle weakness, dyspnea	↑TnT, CK, Mb; ECG; anti-AchR ab	Myocarditis, myositis, rhabdomyolysis, myasthenic crisis	Sintilimab (PD-1i)	NSCLC (adenocarcinoma)	25 days	mPSL, IVIG, PLEX, pyridostigmine; temporary pacemaker; NIPPV → intubation, tracheotomy; antibiotics	favorable
von Itzstein, 2020	♂, 65	Hyperlipidemia, statin-associated myalgia	progressive fatigue, lower extremity weakness, myalgias, diplopia, chest tightness, mild tenderness to palpation of thighs	↑hs-cTn, CK, Mb; ↑urinary Mb; CMR; anti-HMGCR ab, anti-cN-1A ab	Myocarditis (presumptive), myositis (grade 4)	Durvalumab (PD-1I)	NSCLC (squamous, locally advanced)	4 weeks	IVIG, mPSL	favorable
Fazal, 2020	♂, 82	DM2, hypertension	neck stiffness, neck drop, gradually increasing fatigue, dysarthria, unilateral ptosis, weakness of orbicularis oculi, neck muscles, gaze fatigability; orthopnea	↑TnI, CK, AST, ALT;	Myocarditis, myositis, myasthenia	PD-1i	Melanoma (recurrent, metastatic)	2 infusions	IVIG, mPSL; dual antiplatelet; pyridostigmine; Intubation → NIV	fatal
Tomoaia, 2020	♀, 63	N/A	dyspnea, progressive fatigue, lethargy → cardiac arrest (10th day), tachycardia, dry and cold extremities	↑CK, CK-MB, ECG; Echocardiogram; coronary angiography	Myocarditis, myositis, cardiogenic shock, rhabdomyolysis	PD-1i + CTLA-4i	NSCLC (squamous non-keratinizing, metastatic)	6 days	N/A	fatal
Nierstedt, 2020	♂, 77	hypertension, hyperlipidemia, BPN, DVT	tachycardia (110 bpm)	↑Tn; ECG; Echocardiogram	Myocarditis	PD-1i	NSCLC (EGFR+, metastatic)	60 days	mPSL; amiodarone, milrinone, synchronized cardioversion with sedation	fatal
Sato, 2020	♂, 67	N/A	myalgias, high fever	↑TnI, CK, CK-MB; ECG;	Myocarditis	PD-1i + CTLA-4i	RCC (clear cell, metastatic)	20 days	prednisolone, mPSL, IVIG	favorable

				CMR, coronary angiography, EMB					
Tu, 2020	♂, 71	N/A	asymptomatic	↑cTnT, CK-MB, BNP, Mb; CMR (unsuggestive), coronary angiography	Myocarditis (subclinical)	BGB-A317 (experimental anti PD-1; RCT)	NSCLC (poorly differentiated squamous, metastatic)	2 weeks	mPSL	favorable
Fuentes-Antras, 2020	♂, 75	N/A	blurred vision, diplopia, severe asthenia, myalgia, profuse sweating; palpitations	↑TnI, CK, CK-MB, fT4; ECG; anti-AchRs ab, anti-TG ab, anti-TPO ab, anti-striated muscles ab, anti-mitochondria ab, anti-Ro52 ab, anti-SRP ab, anti-PM/Scl100 ab	necrotizing Myocarditis, lymphocytic thyroiditis, necrotizing myopathy, pneumonitis	PD-1i	NSCLC (adenocarcinoma, metastatic)	3 weeks	dexamethasone, methimazole, propranolol; pacemaker; pyridostigmine, mPSL, Infliximab, IVIG; NIV (poorly tolerated)	fatal
Lie, 2020	♂, 79	Asbestos exposure, hypertension, dyslipidemia, stage 3 CKD	severe proximal limb and truncal weakness, dyspnea, fatigue	↑hs-TnT, TnI, CK, NT-proBNP; CMR	Myocarditis (subclinical), myositis	PD-1i	Epithelioid MPM	2 infusions	mPSL; MMF	favorable
Arora, 2020	♂, 70	Hypertension Colon Cancer	palpitations, double vision, right ptosis and pre-syncope	↑TnI, CK, ECG; TTE, CMR	Myocarditis Myasthenia gravis	PD-1i + CTLA-4i	metastatic melanoma	cycle 1, day 12	IV steroids Anti-thymocyte globulin (ATG) mycophenolate mofetil (MMF) and cyclophosphamide Plasmapheresis	arrythmias as cardiac arrest and responded to CPR pacing, no sustained improvement fatal

	♂, 79	CLL	blurred vision, diplopia, fatigue, lower extremity weakness and diffuse pain	ECG - complete heart block, ↑TnI, CK , TTE	Myocarditis, hepatitis Myasthenia gravis	PD-1i	metastatic melanoma		IV steroids ATG and MMF Cyclophosphamide pacemaker	lack of significan t clinical improve ment
	♀, 61	None	right ptosis chest pain, dizziness, dyspnea, arrythmia, ↑QTc	↑TnI, TTE , CMR	Hepatitis Myositis Myocarditis	PD-1i + CTLA-4i	metastatic breast cancer	dose two	IV steroids MMF	declined quickly respirator y failure
	♂, 70	Hypertensi on, CKD, Atrial Fibrillation	generalized weakness and fatigue	↑TnI, CK TTE	Myocarditis MG	PD-1i + CTLA-4i	metastatic kidney cancer	cycle 1 day 21	IV steroids and supportive care After plasmapheresis	Favorable , after clinically deteriorat ed Intubatio n respirator y failure - comfort measures
	♂, 89	Hypertensi on, Hyperlipid emia, CAD, CKD, Type 2 DM	disconjugate gaze, dysphagia, blurred vision and imbalance	↑TnI, CK TTE, MRI	Hepatitis Myocarditis MG	PD-1i	non-small cell lung cancer	10 days after dose 2	IV steroids	non-sustained VT high-degree AVB - comfort measures
Fazel, 2019	♀, 78	hypertensio n, intermittent asthma, prior pulmonary embolism, depression	diffuse rash; diplopia, gaze palsy, bilateral weakness and myalgias of proximal muscles, unsteady gait, decreased distal vibratory sensation	↑TnI, CK, BNP, CRP; anti-striated muscle ab	Myocarditis, myositis, MG	PD-1i + CTLA-4i	Melanoma (metastatic)	5 days	mPSL, IVIG, plasmapheresis	fatal
So, 2019	♀, 55	thymoma, anti-AchR positivity	dyspnea, neck stiffness, myalgia, limb weakness, progressive ophthalmoplegia, ptosis, dysphagia, myasthenic crisis	↑TnT, TnI, CK, CK-MB; ECG; echocardiogram; anti-AchR ab	Myocarditis, MG, necrotizing myopathy	PD-1i	Melanoma (metastatic)	15 days	IVIG, steroid pulse, PLEX; MV	favorable

Sakai, 2019	♂, 74	No coronary risk factors, normal cardiac function	malaise, decreased appetite, severe dyspnea	↑TnI, CK, CK-MB, BNP; ECG; echocardiogram	myocardial necrosis, acute HF	PD-1i	NSCLC (adenocarcinoma, stage IIIB)	1 week	catechocardiography lamines, extracorporeal circulation-assisting device	fatal
Wang, 2019	♂, 45	N/A	tachycardia (110 bpm); fever, hip rashes; left knee pain, weakness, myalgia	↑TnT, NT-proBNP; CK, CK-MB – normal; ECG; Echocardiogram, Coronary angiography, CMR; ANA (1:100)	Myocarditis, pneumonitis	PD-1i	Nasopharyngeal cancer (metastatic)	4 days	moxifloxacin, cefminox (no response); mPSL	favorable
Szuchan, 2019	♀, 70	N/A	dyspnea, orthopnea, hypertension (158/93 mmHg), tachypnea (32 rpm), complete heart block (20-30 bpm), severe respiratory failure; weakness	↑TnT, CK, CK-MB; ECG, echocardiogram, coronary angiography EMB; anti-MuSK ab, anti-AchR ab	Myocarditis, MG ; PTE	PD-1i	Thymoma B3 (metastatic)	3 weeks	MV → BiPAP → NC; enoxaparin, isoproterenol drip, temporary pacemaker; mPSL, plasmapheresis; pyridostigmine	favorable
Valenti-Azcarate, 2019	♂, 66	N/A	diplopia, fatigue, mild dyspnea, upper back pain, limited bilateral eye abduction, mild left ptosis, right trapezius tenderness	↑TnT, CK, aldolase; ECG, echocardiogram, CMR; GFAP-IgG ab	Myocarditis, myositis	PD-1i + CTLA-4i	NSCLC (metastatic)	4 weeks	prednisolone	favorable
Esfahani, 2019	♀, 71	N/A	dyspnea, ptosis	↑hs-TnI, hs-TnT, CK; ECG; CMR; anti-AchR ab	Myocarditis	PD-1i	Melanoma (metastatic)	2 infusions	mPSL, MMF, plasmapheresis, rituximab, alemtuzumab; intubation	favorable
Hyun, 2019	♀, 45	chronic HBV carrier, ocular MG, thymoma	quadriparesis, ptosis, exertional dyspnea, severe orthopnea	↑TnI, CK, CK-MB; ECG; anti-Ach ab	Myocarditis (fulminant), MG crisis, hepatic dysfunction	PD-1i	Thymoma B2 (stage IVA)	15 days	CPR, ECMO, continuous renal replacement therapy (cardio-renal syndrome); mPSL	fatal

Saibil, 2019	♂, 67	controlled hypercholesterolemia hypertension	fatigue, weakness, dyspnea. respirator y distress hypotension fever	↑TnI, CK, Echocardiogram, Coronary angiography, Cardiac catheterization	Myocarditis	CTLA-4i + PD-1i	melanoma	2 weeks	mPSL infliximab IVIG dialysis	fatal
Rota 2019	♂, 71	N/A	dropped head, limb weakness	neurophysiological investigation	Myocarditis, complex neuromuscular disorder	PD-1i	renal cancer		IVIG steroids	fatal
Lie 2019	♂, 79	asbestos exposure, HBP dyslipidemia chronic kidney dis.	proximal limb and truncal weakness, dyspnea and generalized fatigue	↑TnI, CK, negative rheumatological panel markers, ECG muscle biopsy cardiac MRI PET/CT scan 3 months - progression	Myocarditis,	PD-1i	Malignant pleural mesothelioma	2 cycles	1,000 mg IV pulse methylprednisolone	discharged home favorable PET/CT scan - near complete metabolic remission PET/CT
Monge, 2018	♂, 79	AF, QT prolongation, LAFB	blurred vision, upper back pain and stiffness	↑TnI, CK, CK-MB, proBNP; Echocardiogram	Myocarditis, muscular symptoms	PD-1i	mCRPC	8 weeks	mPSL; PROSTVAC continued	favorable
Samara, 2018	♂, 77	Hypertension, mild kidney dysfunction, no history of heart, liver, or autoimmune disease	malaise, nausea, cough, decreased appetite, weight gain, hypotension (84/57mmHg), bradycardia (54 bpm), lower limb edema	↑Tn, ALT, AST, Cr (acute-on-chronic renal failure); ECG; Echocardiogram; anti-smooth muscle ab	Myocarditis, HF, skin rash (grade 2)	CTLA-4i	Melanoma (stage III)	9 weeks	anti-hypertensive medications discontinued; mPSL	fatal
Thibault, 2018	♂, 52	N/A	asymptomatic (first course - high fever, rigors, skin rash)	↑hs-TnIc (mild); CMR	Myocarditis, skin rash (grade 1)	PD-1i + CTLA-4i	RCC (stage IV)	3 weeks	switch to PD-1i alone, BB	favorable
Mahmood, 2018	♀, 75	N/A	progressive exertional dyspnea, bradycardia (40 bpm); neck weakness, imbalance	↑TnI, CK, AST, ALT; ECG; Chest CT, Echocardiogram; cardiac	Myocarditis, skin pruritus (grade 1)	Durvalumab (PD-1i) + Tremelimumab (CTLA-4i)	ESC (pIIIC2)	4 weeks	mPSL; TVP, oxygen therapy, enalapril (for HF)	favorable

				catheterization, EMB						
Yamaguchi, 2018	♂, 60	N/A	hypotension (81/54 mmHg), tachycardia (130 bpm); fatigue, fever	↑TnT, CK; ECG, Echocardiogram, Coronary angiography, EMB	Myocarditis (fulminant)	CTLA-4i → PD-1i	Melanoma (metastatic)	4 infusions (CTLA-4i) + 13 infusions (PD-1i)	prednisolone, IVIG ; inotropes, VA-ECMO, IABP	favorable
Hellman, 2018	♂, 84	AF	bilateral ptosis, gaze palsy, unsteady gait, imbalance, weakness, mild dysphagia; pulseless cardiac arrest (9 days after admission)	↑TnI, CK, CK-MB; ECG; Echocardiogram	Myocarditis, necrotic myopathy/myositis	PD-1i	Urothelial carcinoma (high-grade, recurrent, stage III)	2 infusions	pacemaker, chest tube (PTX), tracheostomy (unable to weaning), NG tube; prednisone → solumedrol	fatal
Martinez-Calle, 2018	♀,67	N/A	dyspnea on minimal exertion	↑Myocardial injury markers; ECG; Echocardiogram, Coronary angiography	Myocarditis myositis	PD-1i	free light-chain lambda multiple myeloma	2 weeks	mPSL +infliximab	fatal
Chang, 2018	♀,47	N/A	chest pain, fever	↑TnI;ECG; Catheterization, Echocardiogram, CMR.	Myocarditis	PD-1i	multiple myeloma	1 day after the last dose of PD-1i	mPSL	favorable
Jain, 2018	♂,67	N/A	back pain<br>skin rash<br>hypotension,<br>acute kidney injury<br>progressive SOB, cough, and dyspnea on exertion	ECG, TTE -> ↑TnI, CK, CK-MB, NT-ProBNP EKG's -new intraventricular conduction delay NSVT; TTE , Coronary angiogram; ECH – 3rd AVB; Right heart catheterization with endomyocardial biopsy	Myocarditis	PD-1i + CTLA-4i	melanoma	6 day -> after 10 day	1 mg/kg of prednisone<br>Pacing<br>Methylprednisolone<br>inotropic and ventilatory support<br>antithymocyte globulin	Before discharge - Deteriorated pursuit of palliative care and inpatient hospice.
Frigeri, 2018	♀, 76	N/A	progressive dyspnea, pulmonary rales,<br>lower limb edema	↑Hs-TnI, NT-proBNP, Echocardiogram,	Myocarditis, cardiogenic shock	PD-1i	lung adenocarcinoma	not mentioned exactly,	Inotropic drugs, ECMO<br>, IABP.	favorable

				CMR, Coronary angiography				(after 7 biweekly administrati ons)	mPSL Plasmapheresis immunoglobulin G infliximab defibrillator	
Tay, 2017	♀, 64	N/A	Diplopia, myalgias, proximal weakness	↑TnI, CK,ECG, TTE Electromyograph y Cardiac biopsy Coronary angiogram Temporary pacing	Myocarditis, Myositis	PD-1i	Glioblastoma	Eight days after her second trial	Iv methylprednisolone Infliximab – single dose Amiodarone Betablocker Day 8- VT , hemodynamic compromise – cardioversion – complete heart block - Temporary pacing ATGM therapy Mycophenolate	Favorable
Chen, 2017	♂, 43	N/A	moderate chest pain, dyspnea, recurrent hypotension, cardiac arrest; generalized myalgias, diplopia, dysphagia	↑TnI, CK, CK-MB, Mb, NT-proBNP, CD8+ LyT; ↓CD4+ LyT; ECG, Echocardiogram; EMB; anti-AchR ab	Myocarditis, rhabdomyolysis	PD-1i	Thymoma B3 (recurrent, metastatic)	10 days	IGIV, mPSL; MV, IABP, hemodialysis, temporary pacemaker	fatal
Tajmir -Riahi, 2017	♂, 72	Old MI, DM2, hypertensio n, PAOD IIB, hyperurice mia	dyspnea, weight gain, limb edema, pleural effusion, ascites	Echocardiogram, CMR, EMB	Myocarditis (2 episodes); autoimmune hyperthyroidism, hypophysitis (previously)	PD-1i + CTLA-4i → PD-1i	Melanoma (metastatic)	22 weeks; 10 months without ICI; 2 weeks after	prednisolone, diuretics	fatal
Masto n, 2017	♂, 55	diabetic ketoacidosi s (24 days after ICI)	Lethargy, shortness of breath	↑TnI; ECG; Echocardiogram, CT, Coronary angiography,	Myocarditis, multiorgan failure, cardiogenic shock	PD-1i	lung adenocarcino ma	7 weeks	not mentioned	fatal

				Cardiac catheterization						
Arangalage, 2017	♀, 35	N/A	progressive dyspnea	↑TnI, CK; ECG, Echocardiogram, CMR	Myocarditis, immune-related thyroid dysfunction	CTLA-4i + PD-1i	melanoma	2 weeks	mPSL IVIG, ECMO Plasma exchanges, tacrolimus	favorable
Norwood, 2017	♀, 49	Mild hyperlipidemia	asymptomatic	↑cTnI, CK-MB, CK; ECG; Echocardiogram, CMR, catheterization, EMB	Myocarditis, thyroiditis	CTLA-4i + PD-1i	melanoma	2 weeks	mPSL prednisone IVIG	favorable
Fukasawa,2017	♀, 69	N/A	general malaise double vision, dyspnea	↑CK, CK-MB, TnI; ECG; Echocardiogram, Coronary angiography, EMB; AChR-ab.	Myocarditis + MG	PD-1i	lung adenocarcinoma	7 days after the 3rd cycle	mPSL, temporary noninvasive positive pressure ventilation, temporary cardiac pacemaker	favorable
Reuben, 2017	♂, 80	CAD (bypass grafted), chronic AF	progressive dyspnea, fatigue	↑ TnI, CK, CK-MB, BNP; ECG; Ecocardiogram	myocarditis, autoimmune hepatitis	CTLA-4i	Melanoma (metastatic)	5 weeks, 2 days	corticosteroids	fatal
Semper, 2016	♂, 75	N/A	acute attacks of dyspnea and chest pain	hsTnT, NT-proBNP, D-dimer ECG, TTE Coronary angiography Cardiac MRI	myocarditis	PD-1i	squamous cell carcinoma of the lung	Three days the 9th cycle of	ACE-inhibitors, beta blockers and diuretics prednisolone	Favorable
Berg, 2017	♂, 66	ipilimumab-related colitis after first infusion	myalgias acute-onset SOB, orthopnea, edema	TnI , CK, CK-MB, NT-pro BNP; ECG, chest RX , angio CT , TTE	myocarditis	CTLA-4i	CMML	No data	Iv furosemide Dopamine methylprednisolone	Hypotension Complete AVB comfort measures fatal

Gibson, 2016	♀, 68	DVT, PE<br>WPW<br>recent angiography - non-obstructive disease	Altered mental status, nausea and vomiting	ECG – RBBB<br>↑ TnI, CK, CK-MB<br>Chest Rx<br>ECG - ectopic beats – VT<br>TTE<br>MRI brain	Myocarditis<br>Hepatitis	PD-1i	stage IV lung adenocarcinoma	two doses - 2 weeks apart- 1 week after second dose	amiodarone infusion<br>glucocorticoids<br>ntravenous methylprednisolone	Palliation<br>Fatal

Abbreviations: Ab, antibody; ACEi, angiotensin-converting-enzyme inhibitors, AchR, acetylcholine receptor, AF, atrial fibrillation; ALT, alanine transaminase, ANA, antinuclear antibodies, ARB, angiotensin receptor blockers; AST, aspartate aminotransferase, AVB, atrioventricular block; β1AR, beta-1-adrenergic receptor; BB, beta-blocker; BiPAP, bilevel positive airway pressure; BNP, brain natriuretic peptide; CAD, coronary artery disease; CC, calcium channel; CK, creatine kinase; CK-MB, creatine kinase – muscle/brain; CMR, cardiovascular magnetic resonance imaging; cN1A, cytosolic 5'-nucleotidase 1A; Cr, serum creatinine; CRP, C-reactive protein; CT, computer tomography; CTLA-4i, cytotoxic T-lymphocyte-associated protein 4 inhibitor; DLST, drug lymphocyte stimulation test; DM2, diabetes mellitus type 2; ECG, electrocardiography; eGFR, estimated glomerular filtration rate; EMB, endomyocardial biopsy; Eo, eosinophils; ESC, esopaheal squamous cell carcinoma; GFAP, glial fibrillary acidic protein, Hb, hemoglobin; HCC, hepatocellular carcinoma; HCV, hepatitis C virus; HCQ, hydroxychloroquine; HMGCR, HMG-CoA reductase; HF, heart failure; HfpEF, heart failure with preserved ejection fraction; IABP, intra-aortic balloon pump; ICD, implantable cardioverter-defibrillator; IV, intravenous; IVIG, intravenous immune globulin; LCNEC, large cell neuroendocrine carcinoma; LDH, lactate dehydrogenasem; LyT, T lymphocytes; Mb, myoglobin; MPM, malignant pleural mesothelioma; mPSL, methylprednisolone; MG, myasthenia gravis; MMF, mycophenolate mofetil; MuSK, muscle-specific kinase; MV, mechanical ventilation; NC, nasal cannula; NG, nasogastric; NIPPV, nasal intermittent positive pressure ventilation; NOR90, nucleolus organizer region; NSCLC, non-small cell lung cancer; NT-pro-BNP, N-terminal pro hormone BNP; PO, per oral; PD-1i, programmed cell death 1 inhibitor; PD-L1i, programmed cell death ligand 1 inhibitor; PLEX, plasmapheresis; PLT, platelet count; PM/SCL, polymyositis/scleroderma; RCC, renal cell carcinoma; RYR, ryanodine receptor; SCS, systemic corticosteroid therapy; SJS, Stevens-Johnson syndrome; SRP, signal recognition particle; TEN, toxic epidermal necrolysis; Tn, troponin; TTS, Takotsubo syndrome, VA ECMO, veno-arterial extracorporeal membrane oxygenation, VT, ventricular tachicardia, WBC, white blood cells count.
